# Pancreas agenesis and fetal growth: a semiquantitative analysis

**DOI:** 10.1530/EC-23-0500

**Published:** 2024-01-29

**Authors:** Mireille N M van Poppel, Christopher J Nolan, Gernot Desoye

**Affiliations:** 1 Institute of Human Movement Sciences, Sport and Health, University of Graz, Graz, Austria; 2 Department of Endocrinology at The Canberra Hospital and the Australian National University School of Medicine and Psychology, Canberra, ACT, Australia; 3 Department of Obstetrics and Gynaecology, Medical University of Graz, Graz, Austria

**Keywords:** insulin, fetus, growth, sex

## Abstract

Pancreas agenesis is a rare condition underlying a variant of permanent neonatal diabetes mellitus. Neonates with this condition are born small for gestational age, but less is known about which components of growth are impacted, the timing of the growth restriction and potential sex differences. Our objective was to assess in which periods in gestation complete pancreas agenesis restricts fetal growth and possible sex differences in susceptibility. Published cases (*n* = 49) with pancreas agenesis providing relevant data (gestational age, fetal sex, birth weight, birth length, head circumference, placental weight) were identified by MEDLINE and secondary literature search covering the years 1950–January 2023. Semiquantitative analysis of these case reports used centiles based on Intergrowth-21 reference charts. Neonates with pancreas agenesis were severely growth restricted; however, median centiles for birth weight, birth length, and head circumference of those born before week 36 were significantly higher compared to those born from 36 weeks. Similar results were found when data were separated by before and from 38 weeks. Head circumference was less affected than birth weight or birth length. No sex differences were found. In conclusion, pancreas agenesis severely restricts fetal length and head circumference in addition to weight growth, with stronger effects evident from 36 weeks of gestation. In addition to the well-known effects of insulin on growth of fetal fat mass, the pronounced effect on birth length and head circumference indicates effects of insulin on fetal lean body growth as well. Lack of power may account for failure to find sex differences.

**Significance statement:**

Neonates with complete pancreas agenesis are born small, but the details of their growth deviation, timing, and potential sex differences remain uncertain. All neonates with pancreas agenesis in our study had reduced birth weight, length, and head circumference, with milder effects in those born before 36 weeks compared to after 36 weeks. This trend persisted when data were separated into before and after 38 weeks, with no discernible sex differences. The absence of the pancreas, and therefore insulin, significantly reduces fetal growth, especially after 36 weeks of gestation. In addition to insulin’s known role in fetal fat mass, our findings suggest it has a substantial influence on birth length and head circumference, underscoring its impact on fetal lean body growth.

## Introduction

Pancreas agenesis (PA) is a very rare condition that causes permanent neonatal diabetes mellitus (PNDM) and pancreatic exocrine insufficiency. It presents most commonly with neonatal hyperglycemia in small for gestational age babies. PA can be (i) partial, commonly with absence of the dorsal pancreas, or (ii) complete, with both dorsal and ventral pancreas missing. PA is caused by genetic abnormalities with an increasing number of transcription factor mutations having been identified, including in *GATA6*,* PDX1*, and *PTF1A* (1, 2, 3).

In cases of complete PA, insulin, a major fetal growth regulator, and C-peptide concentrations are usually below the limit of detection in cord blood. The importance of insulin and other pancreatic hormones for fetal growth through the embryonic and fetal stages of development until delivery is not well understood. Thus, studying the consequences of absence of the pancreas may allow important insights in fetal growth regulation by pancreatic hormones.

So far, mainly case reports or qualitative reviews of the literature on PA have been published. Recently, effects of fetal insulin absence on fetal growth in neonates known to have either recessive absence of the *INS1* gene or genes mutation known to cause PA has been reported (4). In this article we report a semiquantitative analysis of literature data of PA cases, with inclusion only of cases with confirmed complete PA. The aim was to answer the following two research questions:

1. From which gestational age is fetal growth compromised by PA? We hypothesized that the impact of a lack of insulin would be greatest during the last weeks of pregnancy, when in normal pregnancies fetal insulin concentrations increase as reflected by increasing cord blood C-peptide concentrations (5).
2. Are there sex differences in the effect of PA on fetal growth? Both sexes follow different growth trajectories and fetal insulin is associated with length and weight in a sex-specific manner (6, 7).

## Methods

### Data acquisition

A MEDLINE search was performed in early January 2023 using the keywords ‘pancreas’ or ‘pancreatic’ combined with the keywords ‘agenesis’ or ‘aplasia’. Furthermore, bibliographies of retrieved articles were reviewed for additional citations. We only selected cases with confirmed complete PA (no pancreas detected on ultrasound or postmortem). In total 38 reports were found, describing 49 cases of complete PA, including reports from 1969 until 2022. Outcomes of interest were the following parameters: sex, birth weight, birth length, and head circumference. When placental weight and/or a genetic diagnosis were reported, they were also recorded. When data were missing in the case report, authors were contacted and asked to provide missing information. Of the 26 authors contacted, 8 replied, and 4 of them were able to provide additional data.

### Data analysis

To standardize for gestational age at birth and to harmonize the data, birth weight, birth length, and head circumference were transformed into centiles for each week of gestation using Intergrowth-21 reference charts (8). Since the charts are for gestational age of 24 weeks or more, cases born before 24 weeks were excluded (*n* = 3). When fetal sex was unknown, the average of the centiles for males and females was used. When no specific gestational age was reported, only ‘near term’ or ‘at term’ a gestational age of 40 weeks was assumed. To calculate the centiles of placental weight, a published centile chart was used, which began at 24 weeks of gestation (9). The differences between the centiles listed for each gestational age in this table was presumed linear, which enabled us to calculate the specific centile by linear interpolation (8).

The cases were separated into subgroups based on gestational age at birth (<36 weeks, ≥36 weeks and <38 weeks, ≥38 weeks), neonatal sex or genetic diagnosis. Differences between subgroups were tested using the Mann–Whitney *U* test with *P* < 0.05 as significance level.

## Results

Of the 49 case reports of complete pancreas agenesis (2, 3, 10, 11, 12, 13, 14, 15, 16, 17, 18, 19, 20, 21, 22, 23, 24, 25, 26, 27, 28, 29, 30, 31, 32, 33, 34, 35, 36, 37, 38, 39, 40, 41, 42, 43, 44, 45, 46), 43 reported neonatal sex (20 female, 23 male). The pregnancies were all singleton. Of four cases, no data on birth weight, birth length, head circumference, or placenta weight were available, and of three cases, gestational age at birth was unknown, and no centiles could be calculated. The median duration of pregnancy was 37 weeks (range 15–41 weeks). Fifteen neonates were born before 36 weeks of gestation, 10 in week 36 or 37, and 21 offspring from 38 weeks of gestation.

Anthropometric characteristics of the neonates are described in Table 1. Median centiles of birth weight, birth length, and head circumference were 0.5, 0.7, and 13.4, respectively. More detailed information, including method of PA diagnosis and genetic analysis findings of each case, are shown in Table 2.

**Table 1:** Anthropometric characteristics of the neonates (*n* = 49).

Neonatal characteristic	*n*	Median (IQR) or *n* (%)	*n*	Median centile (IQR)
Gestational age at birth, weeks	46	37 (34–39)	−	−
Female sex	43	20 (42%)	−	−
Birth weight, kg	45	1.62 (1.34–1.97)	41	0.5 (0.02–2.6)
Birth length, cm	19	41.0 (40.0–44.0)	19	0.7 (0.0–11.9)
Head circumference, cm	15	31.0 (30.0–32.0)	15	13.4 (0.5–22.3)

IQR, interquartile range.

**Table 2:** Characteristics of the cases found for the analysis.

Author	Reference	Sex	Gestational age (week)	Live birth	Birth weight (g, %)	Birth length (cm, %)	Head circumference (cm, %)	Diagnosis of pancreas agenesis	Genetic mutation	NDM	Syndrome reported
Al-Shammari *et al.*^a^	(33)	M	36	Yes (4 m)	–	–	–	US	Mutation in PTF1A	Yes	Cerebellar agenesis, optic atrophy
Ashraf *et al.*	(2)	F	40	Yes	1610 (0.01)	41 (0)	31 (0.68)	CT and surgical exploration	No mutation in Pdx1 or IPF1	Yes	Atrial and ventricular septal defects, absent gallbladder
Barbarini *et al.*	(25)	F	34	Yes	1570 (5.58)	41 (2.88)	31.6 (65.73)	MRI	No mutation found in chromosome 6, KCNJ11, ABCC8, IPF1, PTF1A, HNF1beta	Yes	Diaphragmatic hernia
Baumeister *et al.*	(20)	F	32	Yes	1010 (0.58)	36 (1.11)	28 (22.26)	Repeated US	NA	No	Absent gallbladder, double outlet right ventricle
Body-Bechou *et al.*	(29)	M	30	No	1530 (71.33)	–	–	PM	Mutation in TCF2	NA	Bilateral multicystic renal dysplasia, bilateral clubfoot
Body-Bechou *et al.*	(29)	M	22	No	511 (NA)	–	–	PM	Mutation in TCF2	NA	Bilateral multicystic renal dysplasia
Bruce and Coutts	(40)	M	37	Yes (2 days)	2340 (8.14)	47 (31.99)	32.2 (24.96)	PM	NA	NA	Agenesis of midgut and superior mesenteric artery
Chao *et al.*^a^	(39)	M	Term	Yes	–	–	–	CT	Mutation in GATA6	Yes	Atrial septal defect, Patent Ductus Arteriosus
Chao *et al.*^a^	(39)	M	Term	Yes	–	–	–	Endoscopic retrograde cholangio-pancreatography	Mutation in GATA6	Yes	Cystic duct, gallbladder absent, mitral valve stenosis and patent ductus arteriosus
Chen *et al.*	(21)	M	39	Yes	2800 (13.16)	–	–	CT	No mutation in PDX1, SOX17, HLXB9, PTF1A, and HNF6	Yes	No
Chen *et al.*	(21)	F	39	Yes	2400 (2.62)	–	–	CT	No mutation in PDX1, SOX17, HLXB9, PTF1A, and HNF6	Yes	No
Chen *et al.*	(21)	M	38	Yes	2300 (2.68)	–	–	US and CT	No mutation in PDX1, SOX17, HLXB9, PTF1A, and HNF6	Yes	No
Cospain *et al.*^a^	(43, 44)	F	15	No	71 (NA)	–	–	PM	Mutation in CNOT1	NA	Dysmorphic features, cleft lip, semi lobar holoprosencephaly, absent corpus callosum
De Franco *et al.*	(44)	F	38	Yes	1340 (0.01)	41 (0.02)	30 (0.52)	US and MRI	Mutation in CNOT1	Yes	Absent gallbladder, lobular holoprosencephaly with dysplastic frontal horns of the lateral ventricles, missing septum pellucidum, broadly joined cella media of the lateral ventricles, and hypoplasia of the corpus callosum
De Franco *et al.*	(44)	M	39	Yes	1900 (0.10)	–	–	US	Mutation in CNOT1	Yes	Mild dysmorphic features
Demirbilek *et al.*	(3)	--	31	Yes	1500 (49.50)	--	--	Pancreatic imaging	Mutation in PTF1A	Yes	Developmental delay
Demirbilek *et al.*	(3)	–	39	Yes	2400 (2.25)	–	–	Pancreatic imaging	Mutation in PTF1A	Yes	No
Demirbilek *et al.*	(3)	–	32	Yes	1200 (3.92)	–	–	Pancreatic imaging	Mutation in PTF1A	Yes	Neonatal cholestatis
Dodge *et al.*	(13)	M	39	Yes (3 days)	2130 (0.4)	50 (66.64)	33 (21.68)	PM (no pancreatic islets present in histological examination)	NA	Yes	NA
Dourov *et al.*	(10)	F	33	Yes (19 days)	1800 (44.55)	42 (22.28)	31 (65.55)	PM	NA	No	Absent gallbladder
Evliyaoglu *et al.*	(37)	F	37	Yes	1900 (0.81)	–	–	US	Mutation in PTF1A	Yes	No
Evliyaoglu *et al.*	(37)	F	37	Yes	1520 (0.06)	–	–	US and MRI	Mutation in PTF1A	Yes	Foramen ovale, pulmomary stenosis
Gabbay *et al.*	(32)	M	37	Yes	1935 (1.00)	43 (0.65)	32 (20.03)	US	Mutation in PTF1 No mutation in KCNJ11, ABCC8, INS, EIF2AK3, FOXP3, GATA4, GATA6, GCK, GLIS3, HNF1B, IER3IP1, PDX1, PTF1A, NEUROD1, NEUROG3, NKX2-2, RFX6, SLC2A2, SLC19A2, STAT3, WFS1, and ZFP57.	Yes	No
Hilbrands *et al.* (also described in De Franco *et al.* 2019)	(30, 44)	F	38	Yes (12 weeks)	1100 (0)	–	–	PM	Mutation in CNOT1 No mutations in IPF1, PTF1A, GATA6, GATA4, HNF1B, and HNF6.	Yes	Missing corpus callosum, immature brain development (semilobar holoprosencephaly), absent gallbladder
Houghton *et al.*	(42)	M	38	Yes	1980 (0.44)	–	–	US	Mutation in PTF1A	Yes	Patent ductus arteriosus and a small atrial septal defect
Houghton *et al.*	(42)	F	37	Yes	2000 (1.54)	–	–	US	Mutation in PTF1A	Yes	No
Howard *et al.*	(15)	M	Term	Yes	1950 (0.06)	48 (11.86)	–	C (absence or a generalized secretory defect of pancreatic islets)	NA	Yes	No
Ito *et al.*	(31)	M	30	No	600 (0)	–	–	PM	Mutation in GL13	NA	Absent gallbladder, thyroidal atrophy, adrenal atrophy, malrotation of intestine, atresia of anus, bilateral hypoplasia of kidney, hypoplasia of tentalia, hypospadia, polysyndactyly, polysplenia
Johnson *et al.*	(41)	M	19	No	221 (NA)	–	–	PM	NA	NA	Caudal regression syndrome, ventricular septal defect
Lemons *et al.*	(14)	–	41	No	1350 (0)	40.3 (0)	30.5 (0.06)	PM	NA	NA	NA
Mehes *et al.*	(12)	F	Near term	Yes (11 days)	1750 (0.02)	–	–	PM	NA	Yes	Absent gallbladder
Nakao *et al.* / Suzuki *et al.*	(27, 28)	F	37	Yes	1353 (0.02)	39.5 (0.01)	30 (1.43)	MRI/CT	Mutation in GATA6	Yes	Diaphragmatic hernia, ventricular septal defect, ductus arteriosus
Raghuram *et al.*	(46)	M	34	Yes	1310 (1.37)	38 (0.12)	30 (13.41)	US and MRI	Mutation in GATA6	Yes	Absent gallbladder, unilateral thyroid lobe agenesis, truncus arteriosus
Salina *et al.*	(26)	M	35	Yes	1620 (1.91)	45 (29.45)	–	CT and MRI	No mutation in KCNJ11, SUR1, GCK, PDX1, PTF1A, SOX9, SOX17, HNF6, HLXB9, HNF4a, NEUROD1, HNF1α and HNF1β	Yes	Atrial septal defect
Samaee *et al.*	(23)	M	40	Yes	1800 (0.02)	41 (0)	30 (0.03)	US and CT	NA	Yes	No
Schwitzgebel *et al.*	(19)	F	40	Yes	2140 (0.23)	44 (0.16)	–	US and CT	Mutation in PDX1	Yes	No
Shaw-Smith *et al.*	(36)	–	34	Yes (4 days)	1240 (0.81)	–	–	PM	Mutation in GATA4	NA	Abnormal white matter development
Sherwood *et al.*	(11)	–	At term	Yes (6 weeks)	1280 (0)	37 (0)	29 (0.01)	PM	NA	NA	NA
Stanescu *et al.*	(35)	F	39	Yes (3 months)	1760 (0.04)	–	–	CT	Mutation in GATA6 No mutation in IPF1.	Yes	Cardiac malformation, hydronephrosis hydroureter, absent gallbladder
Taha *et al.*	(22)	M	35	Yes (11 months)	1700 (2.92)	–	–	US and CT	No mutation in PTF1A and PDX1, KCNJ11 and ABCC8 or chromosome 6	Yes	Dysmorphic features, and recurrent bacterial infections
Thomas *et al.*	(24)	M	37	Yes	1560 (0.11)	–	–	US and CT: small amount of tissue that could represent pancreas or small bowel, small hypoechoic structure in area of pancreatic head. Stool elastase <50 µg/g	Homozygous mutation in the IPF1 No mutation in KCNJ11 or GCK	Yes	No
Verwest *et al.*	(18)	M	At term	Yes	1500 (0)	–	32.5 (5.62)	US, CT, and laparotomy	No mutation in PDX1	Yes	Absent gallbladder, choledochal duct stenosis
Voldsgaard *et al.*	(17)	F	37	Yes (48 h)	1400 (0.03)	43.5 (1.62)	31.5 (16.78)	PM	NA	Yes	No
Weedon *et al.*	(38)	F	39	Yes	2800 (18.92)	–	–	CT	Mutation in PTF1A	Yes	No
Weedon *et al.*	(38)	M	39	Yes	2400 (1.88)	–	–	CT	Mutation in PTF1A	Yes	No
Weedon *et al.* ^a^	(38)	F	–	Yes	–	–	–	MRI	Mutation in PTF1A	Yes	No
Wright *et al.*/ Stoffers *et al.*	(16, 55)	F	41	Yes	1700 (0.01)	44 (0.07)	–	US	Mutation in IPF1 No mutation in ΔF508	Yes	No
Yau *et al.*	(34)	M	37	Yes	1740 (0.32)	–	–	During surgery at 8 months	Mutation in GATA6	Yes	Congenital diaphragmatic hernia, absent gallbladder
Zanfardino *et al.*	(45)	F	34	Yes	1180 (0.46)	40 (0.90)	–	C	NA	Yes	No

^a^Gestational age below 22 weeks and thus not included in Fig. 1.

C, clinical; NA, not assessed; NDM, neonatal diabetes mellitus; PM, postmortem; US, ultrasound;

### Gestational age differences

**Figure 1:**
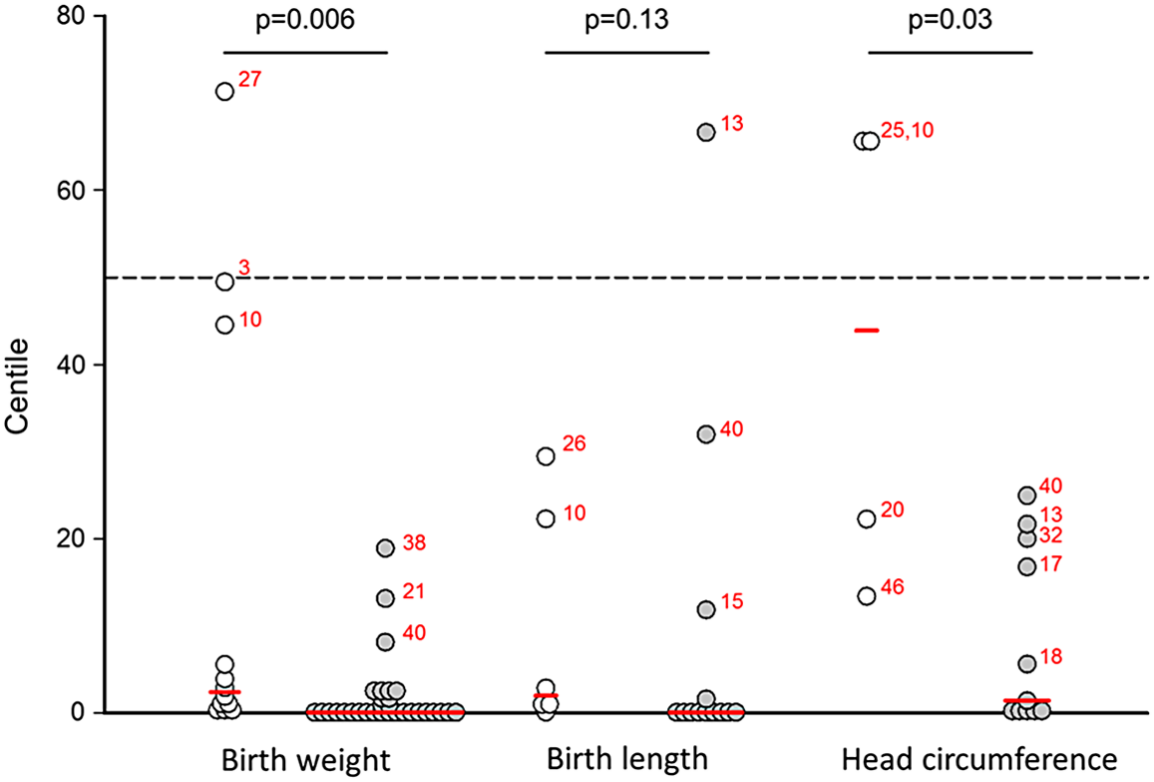
Centiles of birth weight, birth length, and head circumference for neonates born <36 weeks (white circles) and those born ≥ 36 weeks of gestation (gray circles). Red lines indicate median centiles, based on the Intergrowth-21 reference charts (8). The dashed line represents the 50th centile. Red numbers indicate reference where cases/mutation analyses were reported.

In Fig. 1, the median centile for birth weight, birth length, and head circumference of neonates born before week 36 of gestation and those born from 36 weeks are presented. Median centiles for birth weight and head circumference were significantly higher in the neonates born before 36 weeks compared to those born from 36 weeks (2.4 vs 0.1 (*P* = 0.006), 2.0 vs 0.1 (*P* = 0.13), 43.9 vs 1.4 (*P* = 0.03) for birth weight, birth length, and head circumference, respectively). When considering those born before and from 38 weeks gestation, differences in centiles between groups were smaller, but significantly higher in those born earlier for all growth parameters (1.0 versus 0.1 (*P* = 0.03), 1.4 vs 0.01 (*P* = 0.04), 21.2 vs 0.5 (*P* = 0.006) for birth weight, birth length, and head circumference, respectively).

### Sex-specific differences

In the total cohort, no significant sex differences were found in the median centiles for birth weight, birth length, and head circumference (all *P* > 0.15).

### Genetic diagnosis

Genetic analysis data were reported in 37 of 49 cases. For 5 and 11 cases, pancreas agenesis was due to a genetic mutation in the *GATA6* and *PTF1A* gene, respectively, with mutations also being reported in*TCF2*,* PDX1*, *CNOT1*,* GLI3*,* GATA4*, and *IPF1*. No differences with the whole collective were found for birth weight, length, or head circumference. One case with GATA6 and only two with a *PTF1A* mutation were born before 36 weeks of gestation. Hence, the potential influence of specific genetic causes of PA on fetal growth according to gestational age, i.e. before or at and after week 35, could not be assessed. CNOT1 mutations were consistently associated with dysmorphic head and brain features.

### Outlier birth weights

For the five cases with birth weight above the tenth percentile, we checked the diagnosis of pancreas agenesis and genetic analysis findings, and for all cases, the pancreas could not be identified at autopsy or CT/MRI scans (3, 10, 21, 29, 38). Two of the five had a *PTF1A* mutation (3, 37), one a *TCF2* mutation (28), whereas one had no mutation detected and one did not report genetic analysis (9).

### Placental weight

Placental weight was provided in only three cases, all with low placental weight. In the first of these, the placenta weighed 55 g at week 17 (not able to calculate centile), which corresponded to placental weight at week 13 (43). In two other cases, it was 385 g at week 34 (eighth centile) (25) and 480 g at week 37 (12th centile) (17), respectively.

## Discussion

This semiquantitative analysis has assessed the effect of complete PA on growth of human fetuses. The aim was to get more insight into which period of pregnancy the fetus is most dependent on a developing pancreas for growth of the skeleton, the head and overall birth weight, and if there are sex-specific differences. Answering these questions is impossible by qualitatively reviewing reported cases. To make individual data comparable, we have used centiles based on well-established and widely used growth charts.

Our analysis confirms the association of complete PA with severe fetal growth restriction and is consistent with the report of cases with fetal insulin absence determined on the basis of genetic diagnosis (4). While severe growth restriction was evident in the majority of neonates born before 36 weeks gestation, the findings show that fetal growth is exceedingly dependent on the pancreas in the last weeks of pregnancy. Furthermore, the effect of PA is more pronounced on fetal length (i.e. skeleton) and weight growth than on head growth, although head growth is severely impacted in late pregnancy. Sex differences in birth weight, birth length, and head circumference percentiles, which are adjusted for sex using the Intergrowth-21 reference charts, were not detected, which may reflect lack of power and variation in the data, which in turn precluded detecting a difference.

Among the four hormones of the endocrine pancreas, i.e. insulin, glucagon, pancreatic polypeptide hormone and somatostatin, insulin is the key regulator of growth as it acts as a potent mitogen and is anabolic. Growth regulating functions of pancreatic polypeptide hormone and somatostatin have not been found to date. Glucagon may be inhibitory to fetal growth (47). Its absence, therefore, should favor increased fetal size. Thus, growth restriction found in neonates with PA is most likely attributable to the absence of insulin. In infants with Donohue syndrome, a rare genetic disorder characterized by absence of insulin receptors, fetuses are also undergrown (48). The essential role of the pancreas for fetal growth is also shown by experimental pancreatectomy in sheep (49).

Insulin acts on lean body growth either directly, or indirectly by inducing hepatic IGF-1 production and secretion and fat mass accrual through direct action. In normal human pregnancies, cord blood C-peptide, and by inference insulin, levels are relatively low before week 34 of gestation and rise thereafter (5). This might also explain why the effects of PA are more pronounced in fetuses born from 36 weeks of gestation.

The fetal growth restriction in PA is either symmetric, with reductions in fetal length, weight, and head circumference, or asymmetric, with head (i.e. brain) sparing more evident in neonates born before 36 weeks. These findings indicate that insulin is important to both fetal lean (including skeletal) and fat mass growth*.* The few data on head circumference before 36 weeks suggest asymmetric growth restriction in some of these neonates, which may have long term implications such as increased risk for metabolic syndrome and noncommunicable diseases later in life (50). The association of the CNOT1 mutation with abnormal head and brain development warrants further study. Head circumference in cases of CNOT1 mutation was only reported once and was severely reduced, i.e. at 0.5th centile, suggesting smaller brain volume with the risk for neurodevelopmental delay (51).

There were few placenta weights available for analysis; however, the findings would be consistent with a role of fetal insulin in determining final placenta size. This notion is supported by recent genetic data (52, 53) and animal experiments (54).

While severe growth restriction was evident in the majority of the neonates reported to have PA, there were a small number of outliers. As this may have been a consequence of inaccurate diagnosis of PA, we determined the method used to determine PA and assessed the genetic diagnosis information. The methods were robust, and mutations were found in three of the five cases (*TCF2* in one and *PTF1A* in two). Pancreatic hypoplasia as opposed to PA cannot be completely excluded in these cases as an explanation.

So far, fetal insulin has received attention in pregnancies characterized by maternal diabetes or obesity as major contributor to excessive fat accretion. The collective evidence presented here demonstrates the key role of insulin for fetal growth also in pregnancies of women without metabolic disturbances.

We acknowledge that this semiquantitative analysis has limitations due to the paucity of data. We are hoping that future case reports will include information on serial ultrasound assessments of fetal growth, gestational age at birth, birth weight, birth length, head circumference, placental weight, as well as genetic diagnosis. We also encourage all clinicians who have already reported on PA to screen their records for availability of the data used here and share them with us for a future new and better powered analysis of fetal growth in this very rare genetic disorder.

## Declaration of interest

The authors have no conflict of interest related to this work.

## Funding

The study was conducted without funding. The open access was funded by the University of Graz.

## Data availability statement

Data will be available from the corresponding author upon reasonable request.
